# Inhibition of leukotriene B4 receptor 1 attenuates lipopolysaccharide-induced cardiac dysfunction: role of AMPK-regulated mitochondrial function

**DOI:** 10.1038/srep44352

**Published:** 2017-03-14

**Authors:** Meng Sun, Rui Wang, Qinghua Han

**Affiliations:** 1 Department of Cardiology, The First Hospital of Shanxi Medical University, 85 Jiefang South Road, Taiyuan 030001, China

## Abstract

Leukotriene B4 (LTB4)-mediated leukocyte recruitment and inflammatory cytokine production make crucial contributions to chronic inflammation and sepsis; however, the role of LTB4 in lipopolysaccharide (LPS)-induced cardiac dysfunction remains unclear. Therefore, the present study addressed this issue using an LTB4 receptor 1 (BLT1) inhibitor. Administration of LPS to mice resulted in decreased cardiovascular function. Inhibition of LTB4/BLT1 with the BLT1 inhibitor U75302 significantly improved survival and attenuated the LPS-induced acute cardiac dysfunction. During LPS challenge, the phosphorylated AMPK/ACC signaling pathway was slightly activated, and this effect was enhanced by U75302. Additionally, pNF-κB, Bax and cleaved caspase-3 were upregulated by LPS, and Bcl-2, IκB-α, mitochondrial complex I, complex II, and OPA1 were downregulated; however, these effects were reversed by U75302. The results indicated that the BLT1 antagonist suppressed cardiac apoptosis, inflammation, and mitochondrial impairment. Furthermore, the protection provided by the BLT1 inhibitor against LPS-induced cardiac dysfunction was significantly reversed by the AMPK inhibitor Compound C. In conclusion, inhibiting the LTB4/BLT1 signaling pathway via AMPK activation is a potential treatment strategy for septic cardiac dysfunction because it efficiently attenuates cardiac apoptosis, which may occur via the inhibition of inflammation and mitochondrial dysfunction.

Sepsis is a common and lethal syndrome that has high mortality rates; however, specific anti-sepsis treatments are not currently available^1^,^2^,^3^. Moreover, septic shock is a clinical emergency^4^. The cardiovascular system is an important organ system that is frequently compromised by sepsis and affected by septic shock^5^,^6^. Cardiac dysfunction during sepsis has been widely observed; however, the underlying mechanisms are not well understood^5^. Therefore, the basic pathogenesis of septic acute cardiac dysfunction should be investigated because it affects the incidence and course of cardiac injuries. In the current study, we used lipopolysaccharide (LPS)-induced cardiac injury to simulate sepsis. The effects of LPS on the heart were studied^7^.

Leukotriene B4 (LTB4) is a potent inflammatory lipid mediator that isinvolved in the pathophysiology of asthma^8^,^9^,^10^, cystic fibrosis^11^, pulmonary hypertension^12^, hypertension^13^, acute respiratory distress syndrome^14^, and ischemic heart disease^15^. Additionally, leukocyte recruitment mediated by LTB4 and leukotriene B4 receptor type 1 (BLT1) plays an important role during sepsis. LTB4-mediated sterile inflammation has been shown to promote susceptibility to sepsis in type 1 diabetes^16^. Furthermore, serum LTB4 concentrations are elevated during sepsis^17^,^18^,^19^ and may contribute to sepsis-induced injuries to various organs, as well as septic shock and septic death, because these processes can be attenuated by a BLT1 antagonist^20^,^21^,^22^,^23^.Notably, elevated LTB4 contributes to vascular endothelial disorders during sepsis^18^,^19^. Additionally, inhibition of BLT1 reduced myocardial ischemia/reperfusion injury^24^. These data strongly suggest a role for LTB4 and BLT1 in LPS-induced acute cardiac dysfunction. The AMPK signaling pathway is involved in LPS-induced acute cardiac dysfunction. However, whether AMPK is associated with BLT1 is unknown.

In the present study, we explored the effects of LTB4 and BLT1 in a mouse model of LPS-induced acute myocardial injury and investigated the underlying mechanisms. First, an LPS-induced cardiac dysfunction mouse model was established, and we then investigated the therapeutic potential of inhibiting the LTB4 signaling pathway in LPS-induced cardiac dysfunction. Finally, the effects of a BLT1 antagonist on the AMPK signaling pathway, mitochondrial function, inflammation, and apoptosis were studied during LPS-induced cardiac dysfunction.

## Results

### Effect of BLT1 inhibition on LPS-induced cardiac dysfunction

**Figure 1:**
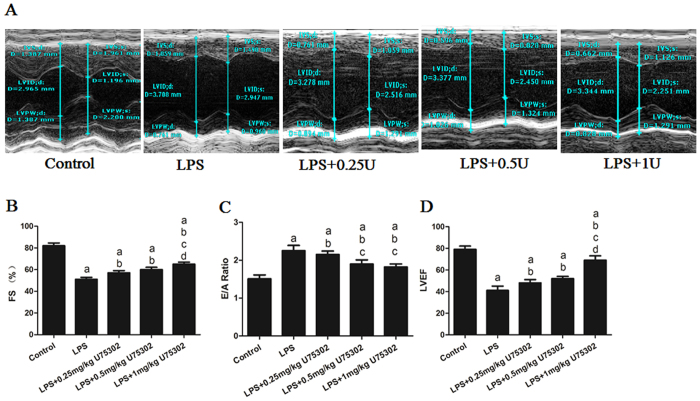
Effect of the BLT1 antagonist U75302 on LPS-induced cardiac dysfunction. PBS and LPS with or without different doses of U75302 was injected intraperitoneal and echocardiographic analysis was conducted 6 h after injection. (**A**) Representative M-mode echocardiograms of mice administered the vehicle, LPS or U75302; (**B**) quantitative analysis of the FS in mice administered the vehicle, LPS or U75302; (**C**) quantitative analysis of the E/A ratio in mice administered the vehicle, LPS or U75302; and (**D**) quantitative analysis of the LVEF in mice administered the vehicle, LPS or U75302. Results are presented as the mean ± SEM (n = 6). ^a^*P* < 0.05 versus control group, ^b^*P* < 0.05 versus LPS group, ^c^*P* < 0.05 versus LPS + 0.25 mg/kg U75302 group, and ^d^*P* < 0.05 versus LPS + 0.5 mg/kg U75302 group. U, U75302.

The mice were intraperitoneally injected with U75302 (0.25, 0.5, or 1 mg/kg) 1 h prior to the administration of 6 mg/kg LPS. Six hours after LPS injection, the mice were anesthetized and subjected to echocardiographic detection. Figure 1A shows representative M-mode echocardiograms for the mice 6 h after LPS injection. The echocardiographic analysis showed that the LPS injection induced a significant decrease in the left ventricular fractional shortening (FS %), left ventricular ejection fractions (LVEF %) and a notable increase in the peak velocity ratios of early to late mitral inflow filling (E/A ratio, Fig. 1B–D, *P* < 0.05). U75302 pretreatment reversed these effects in a dose-dependent manner, with the 1 mg/kg U75302 pretreatment appearing to be the most effective dose (Fig. 1B–D, *P* < 0.05). These data showed that inhibiting BLT1 resulted in the maintenance of cardiac function.

### Effect of BLT1 inhibition on LPS-induced inflammation

**Figure 2:**
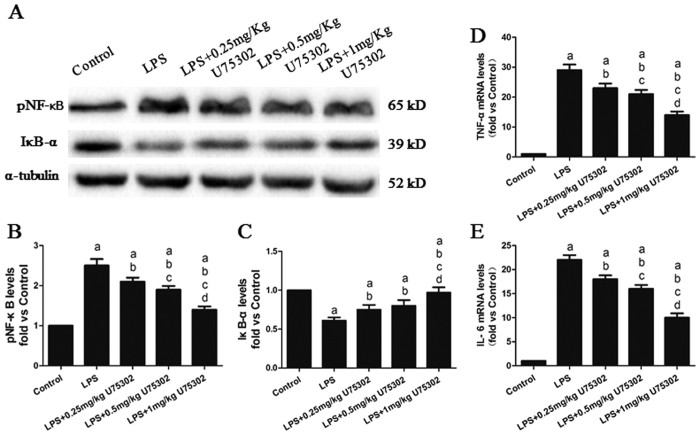
Effect of U75302 on LPS-induced inflammation. PBS and LPS with or without different doses of U75302 was injected intraperitoneal and tissue protein and mRNA were collected 6 h later. (**A**) Representative bands of the western blot; (**B**) pNF-κB expression; (**C**) IκB-α expression; (**D**) TNF-α mRNA levels; and (**E**) IL-6 mRNA levels. The expression levels were calculated by comparing the intensities of the protein of interest and the loading control, followed by standardization. Values are presented as the mean ± SEM (n = 6). ^a^*P* < 0.05 versus control group, ^b^*P* < 0.05 versus LPS group, ^c^*P* < 0.05 versus LPS + 0.25 mg/kg U75302 group, and ^d^*P* < 0.05 versus LPS + 0.5 mg/kg U75302 group.

Firstly, Plasma LTB4 levels were detected 6 h after LPS injection, and LPS treatment significantly increased the plasma LTB4 level from 3.1 ± 0.6 ng/ml to 5.2 ± 1.1ng/ml (P < 0.05). Then we assessed the effect of BLT1 inhibition on LPS-induced inflammation by examining the TNF-α and IL-6 mRNA levels and the pNF-κB and IκB-α protein expression levels in the myocardium. The cardiac levels of TNF-α and IL-6 were assessed by real-time PCR, and the pNF-κB and IκB-α levels were assessed by western blotting (Fig. 2A). LPS treatment significantly increased the TNF-α and IL-6 mRNA levels and pNF-κB expression (Fig. 2B,D,E, *P* < 0.05) but reduced the IκB-α expression (Fig. 2C, *P* < 0.05), whereas U75302 treatment substantially decreased the TNF-α, IL-6 and pNF-κB expression levels and reversed the reduction of IκB-α expression (*P* < 0.05). Another BLT1 inhibitor, CP105, 696, was use to assess the effects of inhibition of BLT1 on LPS-induced inflammation. Compared with the LPS group. CP105, 696 treatment reversed pNF-κB increase and IκB-α decline (Supplementary Fig. 2). These data indicate that BLT1 may play a role in LPS-induced myocardial inflammation.

### Effect of BLT1 inhibition on the LPS-regulated AMPK signaling pathway

**Figure 3:**
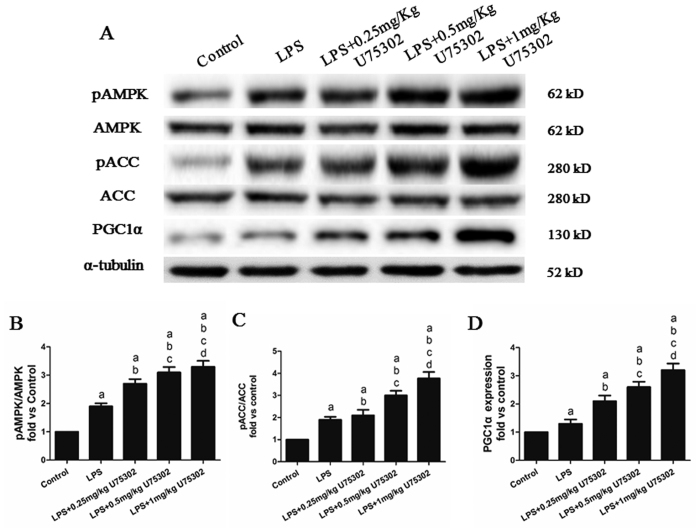
Effects of U75302 on the LPS-regulated AMPK signaling pathway. PBS and LPS with or without different doses of U75302 was injected intraperitoneal and tissue protein were taken 6 h after injection for western blot analysis. (**A**) Representative bands from the western blot analysis. Dose-dependent changes in (**B**) BLT1, (**C**) pAMPK, (**D**) pACC and (**E**) PGC1α in the myocardium. The expression levels were calculated by comparing the intensities of the protein of interest and the loading control, followed by standardization. Values are presented as the mean ± SEM (n = 6). ^a^*P* < 0.05 versus control group, ^b^*P* < 0.05 versus LPS group, ^c^*P* < 0.05 versus LPS + 0.25 mg/kg U75302 group, and ^d^*P* < 0.05 versus LPS + 0.5 mg/kg U75302 group.

To examine the possible involvement of AMPK signaling in the cardiac protective effect of U75302, we assessed the expression of major members of the AMPK pathway (Fig. 3A). After LPS treatment, pAMPK, phosphorylated acetyl-CoA carboxylase (pACC) and PPARγ coactivator-1α (PGC1α) were significantly increased as shown in Fig. 3B–D (the protein phosphorylation levels represent pAMPK and pACC expression relative to AMPK and ACC and were normalized to the mean value of the controls, *P* < 0.05). As expected, inhibiting BLT1 with U75302 resulted in an increase in pAMPK, pACC and PGC1α expression (*P* < 0.05), which indicated further activation of the AMPK signaling pathway. These data showed that inhibiting BLT1 relieved LPS-induced myocardial damage and indicated that this effect may be mediated by the AMPK pathway. To investigate the effect of U75302 treatment alone on the AMPK signaling pathway, we established a control group and a U75302 group, and the expressions of AMPK, ACC, and PGC1α were detected. We found that U75302 had no effect on the AMPK pathway (Supplementary Fig. 1A). The BLT1 expression was also detected to confirm that U75302 is a functional inhibitor (Supplementary Fig. 1A).

### Effect of BLT1 inhibition on LPS-induced myocardial apoptosis

**Figure 4:**
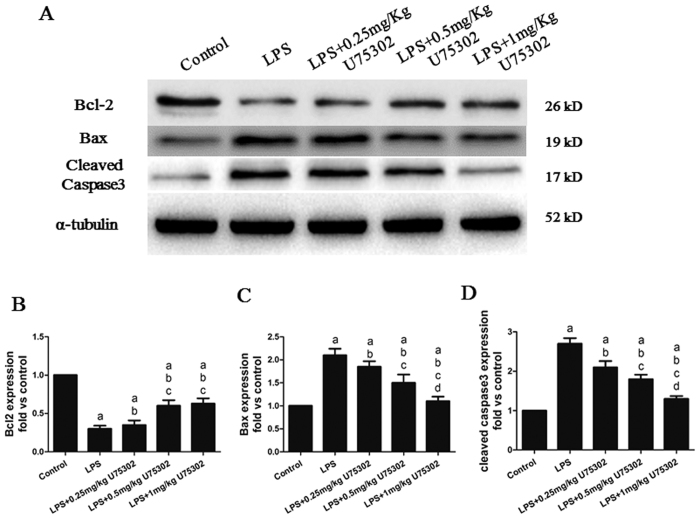
Effects of U75302 on LPS-induced myocardial apoptosis. PBS and LPS with or without different doses of U75302 was injected intraperitoneal and tissue protein were taken for western blot analysis. (**A**) Representative bands of the western blot; (**B**) Bcl-2 expression; (**C**) Bax expression; (**D**) cleaved caspase-3 expression. The expression levels were calculated by comparing the intensities of the protein of interest and the loading control, followed by standardization. Values are presented as the mean ± SEM (n = 6). ^a^*P* < 0.05 versus control group, ^b^*P* < 0.05 versus LPS group, ^c^*P* < 0.05 versus LPS + 0.25 mg/kg U75302 group, and ^d^*P* < 0.05 versus LPS + 0.5 mg/kg U75302 group.

To investigate the role of BLT1 in LPS-induced myocardial apoptosis, we assessed the expressions ofB-cell lymphoma 2 (Bcl-2), Bcl-2-associated X protein (Bax) and cleaved caspase-3 by western blotting. As shown in Fig. 4A, LPS treatment resulted in myocardial apoptosis, whereasU75302 treatment decreased apoptosis in a dose-dependent manner. The LPS treatment reduced Bcl-2 expression and increased Bax and cleaved caspase-3 expression (*P* < 0.05), whereasthe U75302 treatment reversed these changes (Fig. 4B–D, *P* < 0.05). Furthermore, CP105,696 was used to determine the effects of inhibition of BLT1 on LPS-induced apoptosis. CP105, 696 reduced apoptosis through down regulating Bax and increasing Bcl2 (Supplementary Fig. 2).

### Effect of BLT1 inhibition on LPS-induced mitochondrial impairment

**Figure 5:**
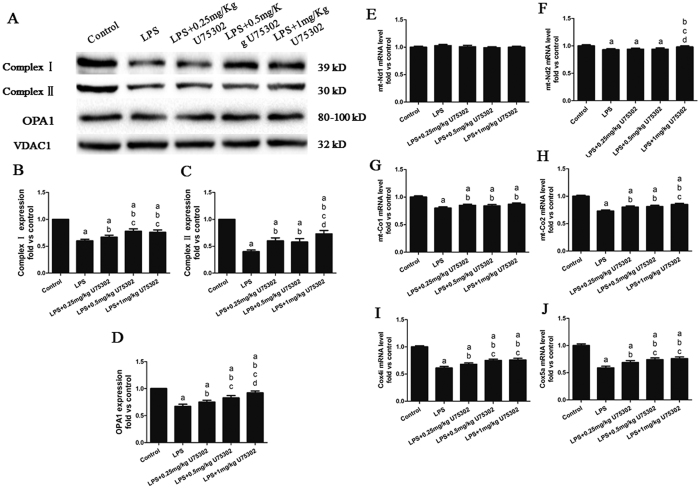
Effects of U75302 on LPS-induced mitochondrial dysfunction. PBS and LPS with or without different doses of U75302 was injected intraperitoneal and tissue protein were taken for western blot analysis. (**A**) Representative bands from western blot analysis; (**B**) mitochondrial complex I expression; (**C**) mitochondrial complex II expression; (**D**) mitochondrial OPA1 expression; and (**E–J**) mRNA levels of the Nd and Co subunits encoded by the nuclear DNA (Cox4i and Cox5a) or mtDNA (mt-Nd1, mt-Nd2, mt-Co1 and mt-Co2). The expression levels were calculated by comparing the intensities of the protein of interest and the loading control, followed by standardization. Values are presented as the mean ± SEM (n = 6). ^a^*P* < 0.05 versus control group, ^b^*P* < 0.05 versus LPS group, ^c^*P* < 0.05 versus LPS + 0.25 mg/kg U75302 group, and ^d^*P* < 0.05 versus LPS + 0.5 mg/kg U75302 group.

To detect the effects of LPS and BLT1 on mitochondrial biogenesis and functions, we examined the expression of mitochondrial complex I, complex II and optic atrophy 1 (OPA1, Fig. 5A)^25^,^26^. As shown in Fig. 5B–D, the LPS treatment led to a reduction in complex I, complex II and OPA1 expression (*P* < 0.05). Next, we evaluated the mRNA expression of the complex I (Nd) and complex IV (Co) subunits encoded by nuclear DNA (Cox4i and Cox5a) or mtDNA (mt-Nd1, mt-Nd2, mt-Co1, and mt-Co2), and we found that LPS induced mtDNA deletion (Fig. 5E, *P* < 0.05). These data indicated that mitochondrial biogenesis and functions were damaged, but U75302 attenuated these changes and maintained complex I, complex II and OPA1 expression (*P* < 0.05). Moreover, U75302 mildly reversed the mRNA changes in the mitochondria (*P* < 0.05). These data indicate that BLT1 may play a role in LPS-induced mitochondrial dysfunction. We determined the purity of the isolated mitochondria using western blotting. As shown in Supplementary Fig. 1B, mitochondrial protein extracts were negative for nuclear, cytosol, endoplasmic reticulum, and cytomembrane markers. In addition, CP105,696 was used to assess the effects of BLT1 inhibition on LPS-induced mitochondrial impairment. In addition, CP105, 696 rescued mitochondrial impairment via maintaining complex I, complex II and optic OPA1 expression (Supplementary Fig. 2).

### Effect of BLT1 inhibition on mouse survival after LPS shock

**Figure 6:**
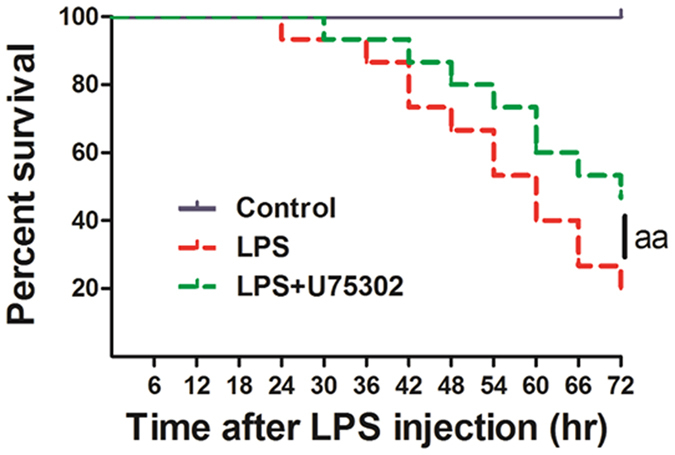
Effects of U75302 on mouse survival after LPS shock. 30 mg/kg LPS was given to mice to assess effects of LPS on survival rate. The mice survival rate was evaluated using Kaplan–Meier curves. ^aa^*P* < 0.01 versus the LPS group (n = 15).

Based on previous data, we selected1 mg/kg as the optimal concentration of U75302. To determine the effects of U75302 on mouse survival after LPS shock, we divided the mice into the control, LPS and LPS + U75302 groups and injected them with PBS, LPS and LPS + U75302, respectively. The mouse survival rate was evaluated using Kaplan-Meier curves (Fig. 6). As expected, mice in the LPS + U75302 group exhibited a significantly lower mortality rate (60.0%) 72 h after LPS injection compared with that of the mice in the LPS group (72.0%) (*P* < 0.01).

### Effect of the AMPK inhibitor Compound C on the U75302-induced protective effects against LPS-induced cardiac dysfunction

**Figure 7:**
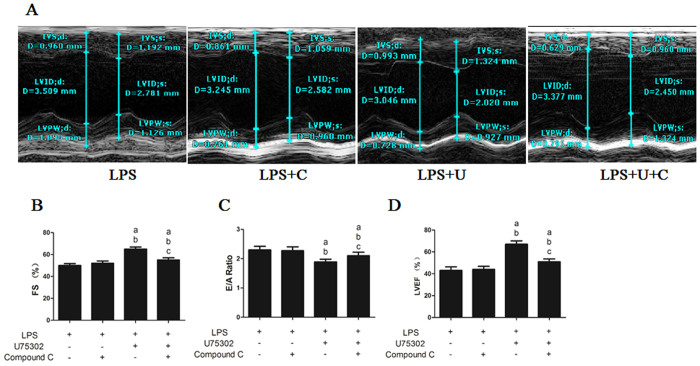
Effects of the AMPK inhibitor Compound C on the protective effect of U75302 in LPS-induced cardiac dysfunction. 6 mg/Kg LPS, 1 mg/kg U75302 and 20 mg/kg Compound C was injected intraperitoneal and echocardiographic analysis was conducted 6 h after injection. (**A**) Representative M-mode echocardiograms of mice administered LPS, U75302 or Compound C; (**B**) quantitative analysis of the FS in mice administered LPS, U75302 or Compound C; (**C**) quantitative analysis of the E/A ratio in mice administered LPS, U75302 or Compound C; and (**D**) quantitative analysis of the LVEF in mice administered LPS, U75302 or Compound C. Results are presented as the mean ± SEM (n = 6). ^a^*P* < 0.05 versus LPS group, ^b^*P* < 0.05 versus LPS + Compound C group, and ^c^*P* < 0.05 versus LPS + 1 mg/kg U75302 group.

Previous data indicated that AMPK signaling might be a downstream pathway of BLT1 in LPS-induced cardiac damage. Therefore, we used the AMPK inhibitor Compound C to examine the role of AMPK in this process. As shown in Fig. 7A–D, Compound C treatment did not result in improvements to the FS, E/A ratio or LVEF compared with that of the LPS group (*P *> 0.05). However, Compound C significantly reduced the FS and LVEF and increased the E/A ratio compared with that of the LPS + U75302 group (*P* < 0.05).

### Effect of Compound C on the U75302-induced protective effects against LPS-induced cardiac inflammation

**Figure 8:**
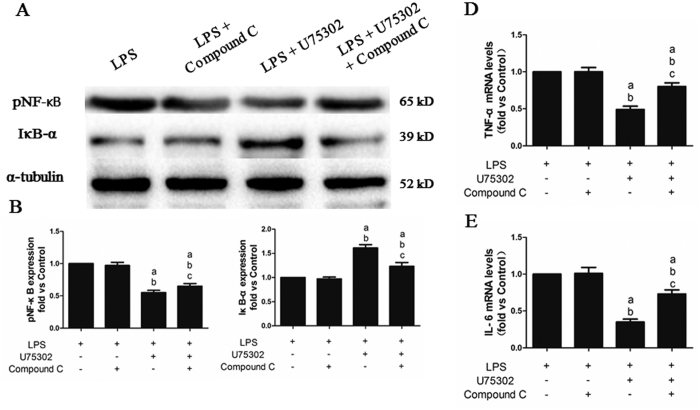
Effects of Compound C on the protective effect of U75302 in LPS-induced cardiac inflammation. 6 mg/Kg LPS, 1 mg/kg U75302 and 20 mg/kg Compound C was injected intraperitoneal and tissue protein and mRNA were taken 6 h later. (**A**) Representative bands of the western blot; (**B**) pNF-κB expression; (**C**) IκB-α expression; (**D**) TNF-α mRNA levels; and (**E**) IL-6 mRNA levels. The expression levels were calculated by comparing the intensities of the protein of interest and the loading control, followed by standardization. Values are presented as the mean ± SEM (n = 6). ^a^*P* < 0.05 versus LPS group, ^b^*P* < 0.05 versus LPS + Compound C group, and ^c^*P* < 0.05 versus LPS + 1 mg/kg U75302 group.

Slight changes were observed in the TNF-α and IL-6 mRNA levels and the pNF-κB and IκB-α protein expression levels after Compound C injection compared with that of the LPS group (Fig. 8A–E, *P > *0.05). As expected, Compound C abolished the anti-inflammatory effect of U75302 by upregulating the TNF-α and IL-6 mRNA levels and pNF-κB protein expression and downregulating IκB-α protein levels (*P* < 0.05).

### Effect of Compound C on the regulation of U75302 following LPS-induced activation of the AMPK pathway

**Figure 9:**
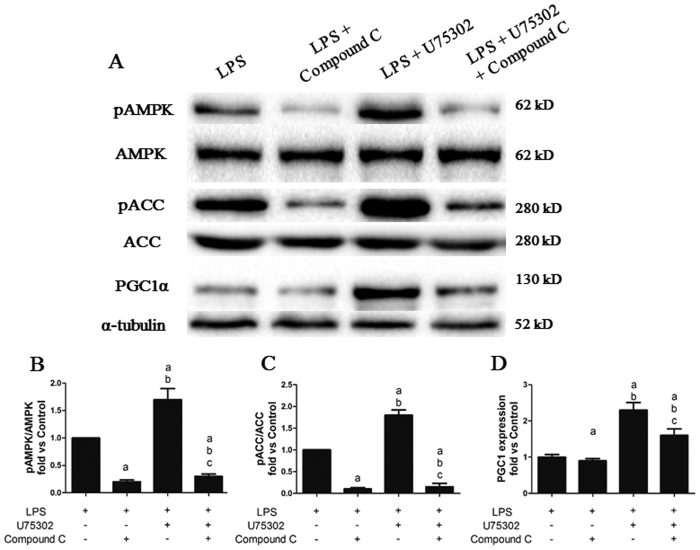
Effects of Compound C on the regulation of U75302 during LPS-induced activation of the AMPK pathway. 6 mg/Kg LPS, 1 mg/kg U75302 and 20 mg/kg Compound C was injected intraperitoneal and tissue protein were taken 6 h later. (**A**) Representative bands from western blot analysis; (**B**) BLT1 expression; (**C**) AMPK phosphorylation level; (**D**) ACC phosphorylation level; (**E**) PGC1α expression. The expression levels were calculated by comparing the intensities of the protein of interest and the loading control, followed by standardization. Values are presented as the mean ± SEM (n = 6). ^a^*P* < 0.05 versus LPS group, ^b^*P* < 0.05 versus LPS + Compound C group, and ^c^*P* < 0.05 versus LPS + 1 mg/kg U75302 group.

Previous data indicated that the BLT1 antagonist U75302 may ameliorate LPS-induced cardiac dysfunction and myocardial damage by activating the AMPK pathway. To test this hypothesis, we measured the expression of key members of the AMPK signaling pathway, including pAMPK/AMPK, pACC/ACC and PGC1α. As shown in Fig. 9A–D, the AMPK inhibitor Compound C significantly suppressed the phosphorylation of AMPK and ACC and mildly downregulated PGC1α expression (*P* < 0.05). Moreover, Compound C abolished the upregulation of PGC1α expression and the phosphorylation of AMPK and ACC induced by U75302 (*P* < 0.05).

### Effects of Compound C on the protective effect of U75302 on LPS-induced myocardial apoptosis

**Figure 10:**
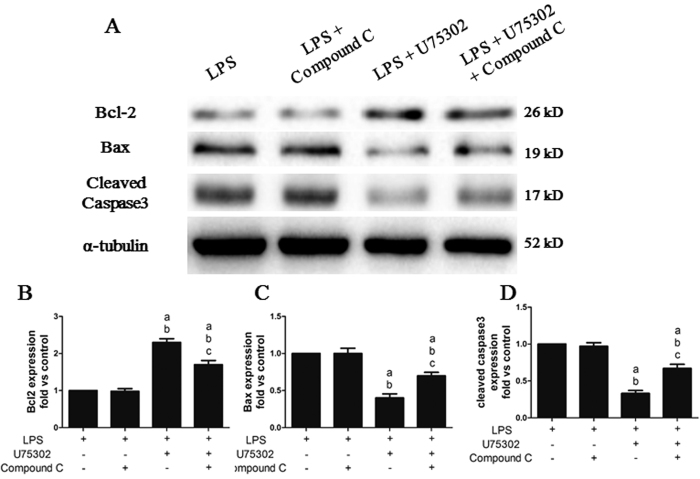
Effects of Compound C on the protective effect of U75302 in LPS-induced myocardial apoptosis. 6 mg/Kg LPS, 1 mg/kg U75302 and 20 mg/kg Compound C was injected intraperitoneal and tissue protein were taken 6 h later. (**A**) Representative bands of the western blot; (**B**) Bcl-2 expression; (**C**) Bax expression; (**D**) cleaved caspase-3 expression. The expression levels were calculated by comparing the intensities of the protein of interest and the loading control, followed by standardization. Values are presented as the mean ± SEM (n = 6). ^a^*P* < 0.05 versus LPS group, ^b^*P* < 0.05 versus LPS + Compound C group, and ^c^*P* < 0.05 versus LPS + 1 mg/kg U75302 group.

As shown in Fig. 10A–D, Compound C treatment did not affect the expression of the apoptosis-related proteins (*P *> 0.05). However, Compound C abolished the increase in Bcl-2 and the reduction in Bax and cleaved caspase-3 induced by U75302 (*P* < 0.05).

### Effects of Compound C on the protective effect of U75302 on LPS-induced mitochondrial impairment

**Figure 11:**
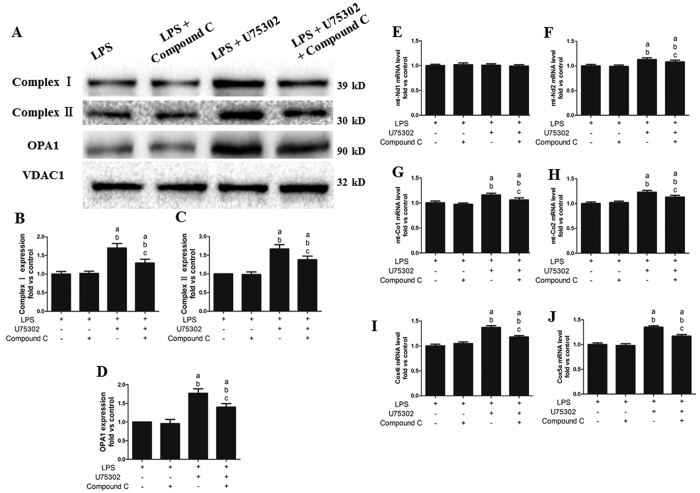
Effects of Compound C on the protective effect of U75302 in LPS-induced mitochondrial dysfunction. (**A**) Representative bands of complex I, complex II and OPA1; (**B**) mitochondrial complex I expression; (**C**) mitochondrial complex II expression; (**D**) mitochondrial OPA1 expression; and (**E–J**) mRNA levels of the Nd and Co subunits encoded by nuclear DNA (Cox4i and Cox5a) or mtDNA (mt-Nd1, mt-Nd2, mt-Co1, and mt-Co2). The expression levels were calculated by comparing the intensities of the protein of interest and the loading control, followed by standardization. Values are presented as the mean ± SEM (n = 6). ^a^*P* < 0.05 versus LPS group, ^b^*P* < 0.05 versus LPS + Compound C group, and ^c^*P* < 0.05 versus LPS + 1 mg/kg U75302 group.

Previous data indicated that BLT1 may mediate LPS-induced mitochondrial damage. To investigate the role of the AMPK signaling pathway in this process, we examined the expression of complex I, complex II and OPA1. As shown in Fig. 11A–D, Compound C pretreatment did not influence complex I, complex II and OPA1 expression compared with that of the LPS group (*P *> 0.05). However, Compound C pretreatment downregulated complex I, complex II and OPA1 expression compared with that of the LPS + U75302 group (Fig. 11A–D, *P* < 0.05). The mt-Nd1, mt-Nd2, mt-Co1, mt-Co2, Cox4i and Cox5a mRNA levels showed a consistent trend in the western blotting results (Fig. 11E). We determine the purity of the isolated mitochondria through western blot analysis (Supplementary Fig. 1B).

### Effects of LTB4 treatment on cardiac function

10 mg/kg LTB4 dissolved in PBS was injected intraperitoneally. 6 h later the echocardiographic analysis was conducted. The FS and EF were markedly lower than that in group control, indicating LTB4 responsible for myocardial injury (P < 0.01). In addition, mice in group LPS + LTB4 showed a notably decreased FS AND EF than that in group LPS (P < 0.05). However, compared with that in group control, mice in group U75302 + LTB4 showed slightly lower EF.

## Discussion

Various studies have demonstrated important roles for BLT1 and LTB4 in sepsis. Type 1 diabetes mellitus (T1DM) is associated with enhanced susceptibility to systemic bacterial infection. However, T1DM mice lacking the receptor for LTB4 survived polymicrobial sepsis, exhibited reduced proinflammatory cytokine production, and had decreased bacterial counts, indicating a role for LTB4 in the enhanced susceptibility of T1DM patients to sepsis^16^. The serum levels of LTB4 were significantly higher in patients who died than in those who survived, indicating that LTB4 could act as an indicator of illness severity^19^,^23^. Moreover, the elevated LTB4 level in septic shock patients may be involved in the deterioration of the pathological features associated with sepsis^17^,^18^. LTB4 was shown to be an important mediator of acute lung and liver injury in a porcine model of sepsis^20^,^21^, and LTB4 was suggested to increase proinflammatory cytokine production and leukocyte recruitment towards the infection site during sepsis^21^,^22^. Furthermore, mortality rates of mice after administration of a BLT1 antagonist were significantly lower than those after administration of LTB4 during polymicrobial sepsis^21^.

Elevated serum LTB4 has also been suggested to induce damage to vascular endothelial cells in patients with sepsis, thus resulting in serious illness^19^. The correlation between LTB4 and vascular endothelial disorders in sepsis patients has been supported by another study^18^. The importance of BLT1 and LTB4 in the pathological changes of the cardiovascular system has also been demonstrated in several studies. Neutrophils from patients with stable angina exhibited substantially increased chemotactic activity and LTB4 generation compared with age-matched control subjects^15^. Furthermore, increased LTB4 generation by neutrophils was observed in patients with unstable angina or acute myocardial infarction compared with patients with stable angina^15^. Atherogenesis induced by intermittent hypoxia was also mediated by LTB4 and BLT1^27^. Activation of the LTB4/BLT pathways by 5-lipoxygenase (a critical enzyme for LTB4 synthesis) leads to atherosclerotic plaque instability^28^,^29^. Therefore, selective BLT1 inhibition may be a promising strategy for preventing myocardial ischemia-reperfusion (IR) injury^30^. The current study found that administering LPS decreased the FS (%) and LVEF (%), increased the E/A ratio, and increased the mortality rate, which can be reversed by U75302 pretreatment. These results indicate that inhibiting the LTB4/BLT1 signaling pathway contributed to the recovery of cardiac tissue after LPS-induced acute cardiac dysfunction.

AMP-activated protein kinase (AMPK) is an important enzyme for the regulation of cellular energy homeostasis. Reports have shown that the AMPK signaling pathway is activated by various factors, including omentin, adiponectin, and mitochondrial aldehyde dehydrogenase, and contributes to cardioprotection against ischemic injury^31^,^32^,^33^ and diabetes-induced myocardial dysfunction^34^,^35^. Furthermore, an AMPK-dependent mechanism may be involved in protection against sepsis-induced organ injury, including the liver and kidney, by decreasing inflammatory cytokines and endothelial activation^36^. AMPK activation also protects against LPS-induced acute cardiac dysfunction^33^. Furthermore, AMPK activation confers protection against cardiac apoptosis and ultimately improves cardiac structure and function^31^,^32^,^33^,^34^. In the current study, the BLT1 antagonist was cardioprotective against LPS-induced acute cardiac apoptosis and dysfunction via the activation of the AMPK signaling pathway, as shown by elevated pAMPK and pACC. This protection was attenuated by an AMPK inhibitor, indicating the important role of AMPK in this process.

Apoptosis is the process of programmed cell death that occurs in various disorders. Apoptosis promoted by challenge with LPS contributes to septic cardiac dysfunction^37^,^38^,^39^. LPS-induced injuries and disorders may be caused by transcription factor nuclear factor-kappa B (NF-κB) activation as well as inflammation^40^,^41^,^42^.Protection against LPS-induced cardiac apoptosis and dysfunction is associated with the inhibition of NF-κB activity and accompanied by reduced production of myocardial proinflammatory cytokines, such as interleukin (IL)-1-beta, IL-6, and tumor necrosis factor (TNF)-alpha^39^,^43^. Furthermore, LPS administration induces mitochondrial dysfunction^25^, which is accompanied by cardiac apoptosis^38^. Studies have suggested that mitochondrial dysfunction induces a major signaling cascade that triggers cell apoptosis^44^,^45^. Collectively, LPS-induced cardiac apoptosis and dysfunction may be caused by activation of NF-κB and mitochondrial dysfunction, which may be associated with AMPK. The disruption of AMPK may have causal roles in mitochondrial dysfunction^46^,^47^. Additionally, AMPK activation inhibits NF-κB signaling^48^,^49^. Notably, LTB4 induced NF-κB activation and augmented proinflammatory cytokine secretion in a BLT1-dependent manner^50^. Furthermore, the BLT1 antagonist reduced inflammation and apoptosis following myocardial IR^30^.In our study, U75302 pretreatment was shown to increase Bcl-2 and decrease Bax and cleaved caspase-3; increase PGC1α, complex I, complex II and OPA1; and increase IκB-α and inhibit pNF-κB, TNF-α and IL-6. These results show that inhibition of BLT1 protects against LPS-induced acute cardiac apoptosis, inflammation and mitochondrial dysfunction, which are consistent with previous studies under other conditions.

**Figure 12:**
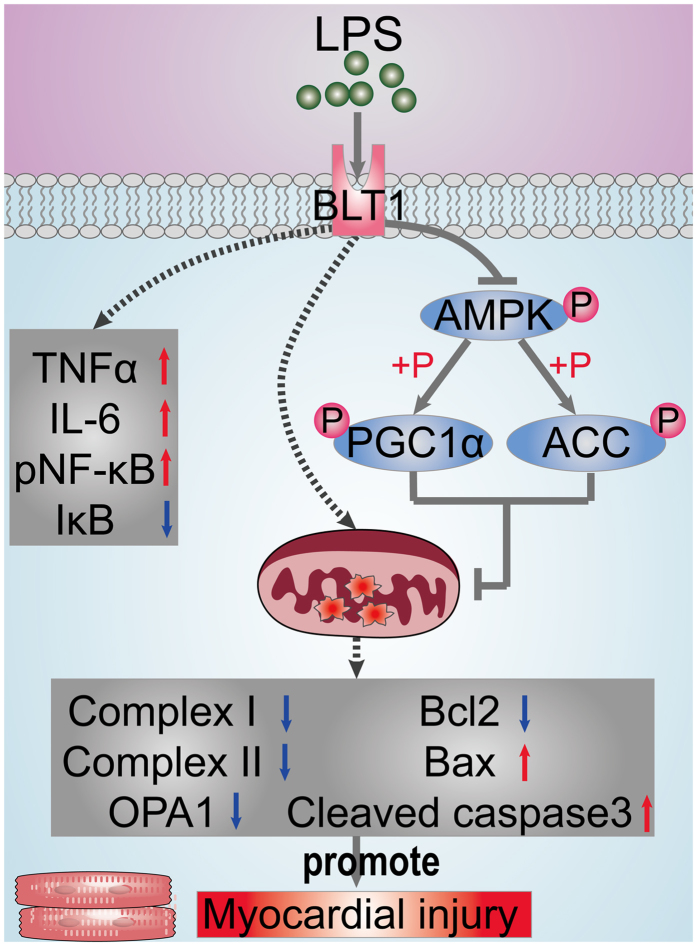
Inhibition of leukotriene B4 receptor 1 attenuates LPS-induced cardiac dysfunction: role of AMPK-regulated mitochondrial function.

In conclusion, our results indicated that inhibition of LTB4/BLT1 reduced cardiac apoptosis via the activation of AMPK, which may have been mediated through the inhibition of NF-κB signaling and mitochondrial impairment (summarized in Fig. 12).

## Methods

### Materials

U75302 (6-(6-(3-hydroxy-1E,5Z-undecadienyl)-2-pyridinyl)-1,5-hexanediol) was purchased from Cayman (Ann Arbor, MI,USA). CP105,696 was purchased from Pfizer (Sandwich, UK). LPS from *Escherichia coli* serotype O55:B5 and Compound C were purchased from Sigma-Aldrich Co. (St Louis, MO, USA). Anti-BLT1 antibody was purchased from Abcam (Cambridge, UK), The anti-pNF-κB, anti-IκB-α, anti-α-tubulin, anti-AMPK, anti-pAMPK (Thr 172), anti-PGC1α, anti-Bcl-2, anti-Bax, anti-cleaved caspase-3, anti-ACC, anti-pACC (Ser 79), anti-calnexin, anti-glyceraldehyde-3-phosphate dehydrogenase (GAPDH), anti-Na+/K+ -ATPase, anti-Histone H3 and anti-VDAC1 antibodies were purchased from Cell Signaling Technology (Beverly, MA, USA). The anti-complex I and anti-complex II antibodies were purchased from Invitrogen Co. (Carlsbad, CA, USA). Anti-OPA1 antibody was purchased from BD Pharmingen (San Diego, CA, USA). Prime Script RT Master Mix, SYBR Premix Ex Taq II and primers were purchased from TaKaRa Co. (Dalian, Liaoning, China). The goat anti-rabbit and goat anti-mouse secondary antibodies were purchased from Zhongshan Co. (Beijing, China). AVEVO 770 high-resolution *in vivo* imaging system was purchased from Visual Sonics Co. (Toronto, ON, Canada). A Bio-Rad imaging system for western blotting was purchased from Bio-Rad (Hercules, CA, USA). A Tissue Mitochondria Isolation Kit was purchased from Beyotime Co. (Nanjing, Jiangsu, China). A TRIzol total RNA extraction kit was purchased from TIANGEN Biotechnology (Beijing, China).

### Animals

The study protocols were approved by the Fourth Military Medical University Committee on Animal Care, and all experiments were performed in accordance with the National Institutes of Health Guidelines for the Use of Laboratory Animals. Male C57 mice weighing 20 g to 22 g (10–12weeks old) were used. The mice were housed under a 12/12-h light/dark cycle with free access to regular rodent chow and tap water.

### LPS-induced acute cardiac injury model

On the day of the experiment, the mice were injected intraperitoneally with a single dose of phosphate-buffered saline (PBS, control group) or LPS dissolved in PBS (LPS group). To generate the Kaplan–Meier survival curve, the C57 mice were divided into the control group (n = 15), LPS group (n = 15) and LPS + U75302 group (n = 15) and injected intraperitoneally with PBS, 30 mg/kg LPS, or 30 mg/kg LPS + 1 mg/kg U75302, respectively. U75302, a specific BLT1 antagonist, was administered for 1 h prior to the LPS injection. The mice were monitored for lethality every 6 h for 3 days. In other experiments, LPS was injected intraperitoneally at a dose of 6 mg/kg. In several experiments, the AMPK inhibitor Compound C was injected intraperitoneally at a dose of 20 mg/kg 30 min prior to the LPS injection. The usage and dosage of U75302 and Compound C were discussed in previous studies^32^,^51^.We administered 3 mg/kg CP105,696 1 h before the LPS injection.

### Echocardiographic analysis

Six hours after the LPS intraperitoneal injection, the mice were subjected to transthoracic echocardiography to assess their cardiac geometry and function using an Esaote Twice System with a SL3116 transducer. Two-dimensional and M-mode echocardiographic measurements were performed with a VEVO 770 high-resolution *in vivo* imaging system. We quantified the left ventricular ejection fraction (LVEF) and left ventricular fractional shortening (FS) from the M-mode images. The ratio of the peak velocity of early to late filling of the mitral inflow (E/A ratio) in the mice was quantified based on Doppler imaging.

### Assessment of mRNA expression by quantitative real-time PCR

Six hours after LPS injection, total RNA was extracted from the left ventricular tissue using a TRIzol total RNA extraction kit. Then, complementary DNA (cDNA) was generated from 500 ng of total RNA by reverse transcription using the Prime Script RT Master Mix. Quantitative real-time reverse transcriptase PCR analyses using SYBR Premix Ex Taq II were performed to detect the TNF-α and IL-6 mRNA levels. The forward and reverse primer sequences, 5′-ACCACCATCAAGGACTC-3′ and 5′-TGACCACTCTCCCTTTG-3′, respectively, were used for TNF-α, and the forward and reverse primer sequences,5′-TTCCAATGCTCTCCTAACAG-3′ and 5′-CTAGGTTTGCCGAGTAGATC-3′, respectively, were used for IL-6. The expression levels of the transcripts were compared to those of α-tubulin and normalized to the mean value of the controls.

### Western blotting

Myocardial tissues were harvested 6 h after LPS injection and used to prepare protein samples containing equal amounts of protein as previously described^52^. For several experiments, we extracted tissue mitochondria using a Tissue Mitochondria Isolation Kit (Beyotime). The proteins were separated by SDS-PAGE electrophoresis and transferred to nitrocellulose membranes. The membranes were blocked with 5% fat-free milk for 2 h in Tris-buffered saline and Tween 20 (TBST, pH 7.6) and incubated overnight at 4 °C with the appropriate primary antibodies [anti-pNF-κB, anti-IκB-α, anti-α-tubulin, anti-AMPK, anti-pAMPK (Thr 172), anti-PGC1α, anti-Bcl-2, anti-Bax, anti-cleaved caspase-3, anti-ACC, anti-pACC (Ser 79), anti-VDAC1, anti-complex I, anti-complex II and anti-OPA1]. The membranes were washed and incubated with the appropriate secondary antibodies. The bands were visualized with a Bio-Rad imaging system and quantified using the Image Lab software package (Bio-Rad). Protein expression levels were compared to that of α-tubulin and normalized to the mean value of the controls.

### Cardiac mitochondria extraction

A Tissue Mitochondria Isolation Kit (Beyotime Technology, China) was used in this experiment. Hearts were washed in PBS and minced in 1 ml of PBS. Then, the tissue was centrifuged at 600 g for 15 s (4 °C). The pellet fraction obtained was digested by 800 μl of 0.25% trypsin for 20 min and centrifuged at 600 g for 15 s (4 °C). The pellet fraction was washed with 200 μl of solution A and centrifuged at 600 g for 15 s (4 °C). Next, the pellet fraction was homogenized and centrifuged at 600 g for 5 min (4 °C). Then, the supernatant fraction was centrifuged at 11,000 g for 10 min (4 °C). The final pellet fraction was the mitochondria.

### Statistical analysis

All of the values are presented as the mean ± standard error of the mean (SEM). The statistical significance of each variable was evaluated by one-way ANOVA. All of the groups were analyzed simultaneously using the LSD t-test, and the survival curves (Kaplan-Meier curve) were analyzed using the log-rank test. *P* values less than 0.05 were considered statistically significant.

## Additional Information

**How to cite this article:** Sun, M. *et al*. Inhibition of leukotriene B4 receptor 1 attenuates lipopolysaccharide-induced cardiac dysfunction: role of AMPK-regulated mitochondrial function. *Sci. Rep.* **7**, 44352; doi: 10.1038/srep44352 (2017).

**Publisher's note:** Springer Nature remains neutral with regard to jurisdictional claims in published maps and institutional affiliations.

## Supplementary Material

Supplementary Information
