# Supplementary material for: Inhibition of leukotriene B4 receptor 1 attenuates lipopolysaccharide-induced cardiac dysfunction: role of AMPK-regulated mitochondrial function
Source: Sci Rep. 2017 Mar 14;7:44352. doi: 10.1038/srep44352 (PMC5349523; doi:10.1038/srep44352)

Supplementary Information

Title of manuscript:

Inhibition of leukotriene B4 receptor 1 attenuates lipopolysaccharide-induced cardiac dysfunction: role of AMPK-regulated mitochondrial function

Authors:

Meng Sun, Rui Wang, Qinghua Han

Supplementary information includes:

Supplementary Figs. S1-S11

Supplementary Fig. 1 Effect of U75302 treatment on the expression of BLT1 and AMPK signaling pathway as well as the mitochondria purity detection A. Representative bands of the western blot of BLT1, pAMPK, AMPK, pACC, ACC, PGC1α; The expression levels were calculated by comparing the intensities of protein of interest and the loading control, followed by standardization. Values are presented as the mean ± SEM (n = 6); **B.** Purity of mitochondria was confirmed by detecting the Histone H3 (nuclear), GAPDH (cytosol), calnexin (endoplasmic reticulum), VDAC1 (mitochondria) and Na^+/K^+-ATPase (cytomembrane) of each simple. C. Plasma LTB4 levels. Values are presented as the mean ± SEM (n = 6). $^{**}P < 0.01$ versus Control group; D. Effects of LTB4 on cardio function. 10mg/kg LTB4 was given and echocardiographic analysis was conducted 6 h later. Values are presented as the mean ± SEM (n = 6). $^{aa}P < 0.01$ versus Control, $^{b}P < 0.05$ versus LPS group, $^{cc}P < 0.01$ versus LTB4 group, $^{c}P < 0.05$ versus LTB4 group.

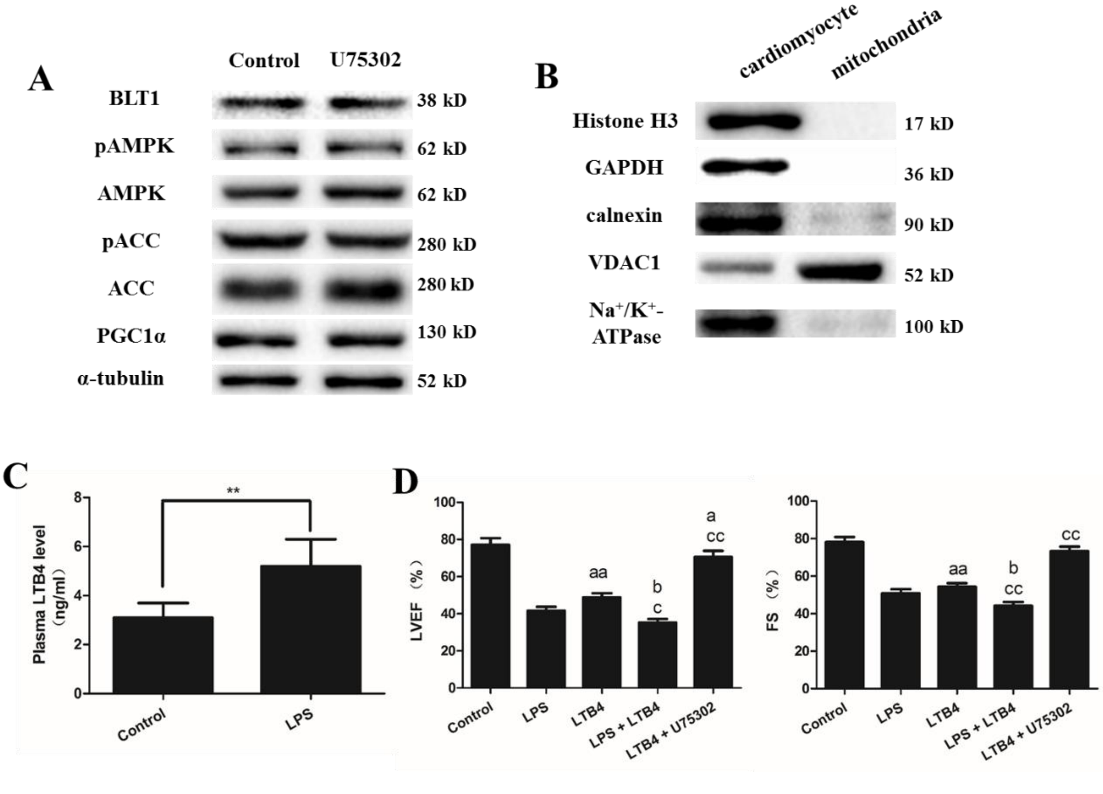

Supplementary Fig. 2 Effect of U75302 on LPS-induced inflammation, myocardial apoptosis and mitochondrial dysfunction. A. Representative bands of the western blot of pNF-κB, IκB-α, Bcl-2, Bax, Complex Ⅰ, Complex Ⅱ and OPA1; B. Expressions of pNF-κB, IκB-α, Bcl-2, Bax, Complex Ⅰ, Complex Ⅱ and OPA1. The expression levels were calculated by comparing the intensities of protein of interest and the loading control, followed by standardization. Values are presented as the mean ± SEM (n = 6); [a]P < 0.05 versus control group, [b]P < 0.05 versus LPS group, [c]P < 0.05 versus CP105,696 group.

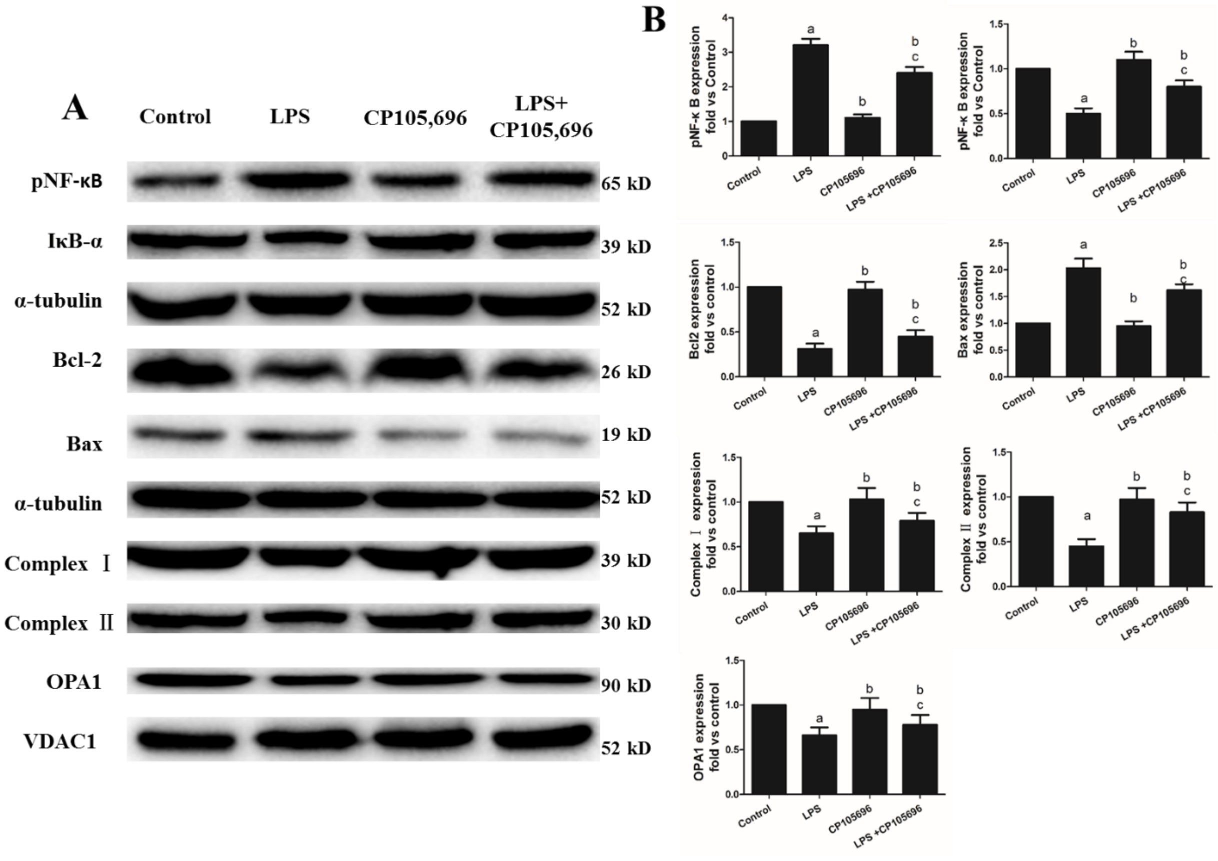

Supplementary Fig. 3 The full length blots in Fig. 2A

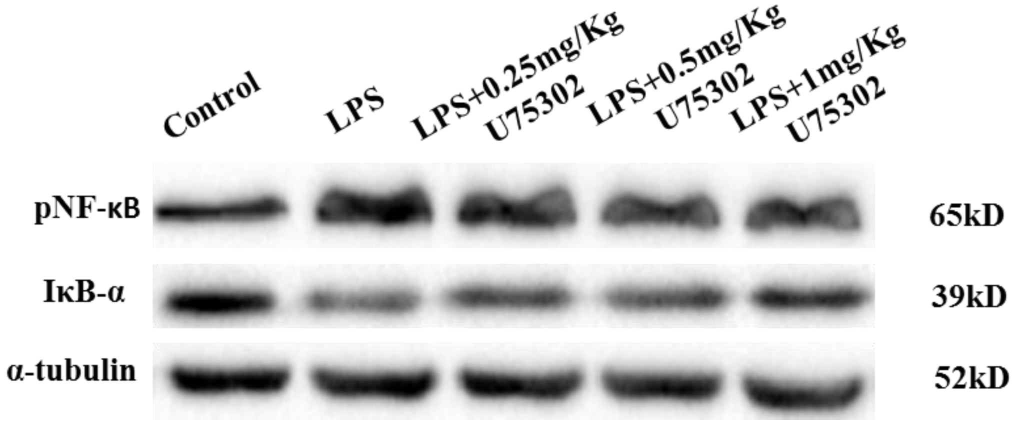

Supplementary Fig. 4 The full length blots in Fig. 3A

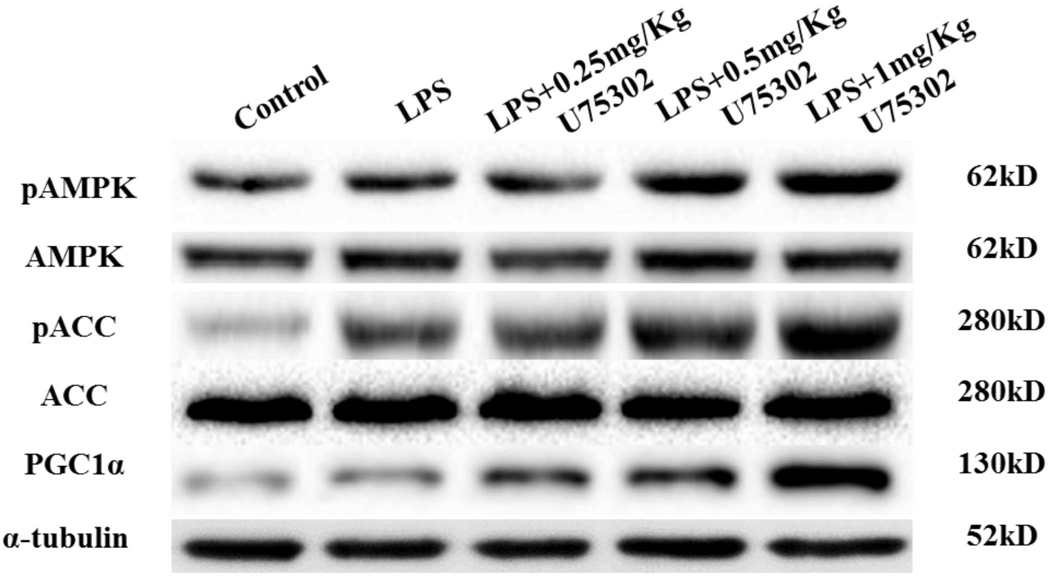

Supplementary Fig. 5 The full length blots in Fig. 4A

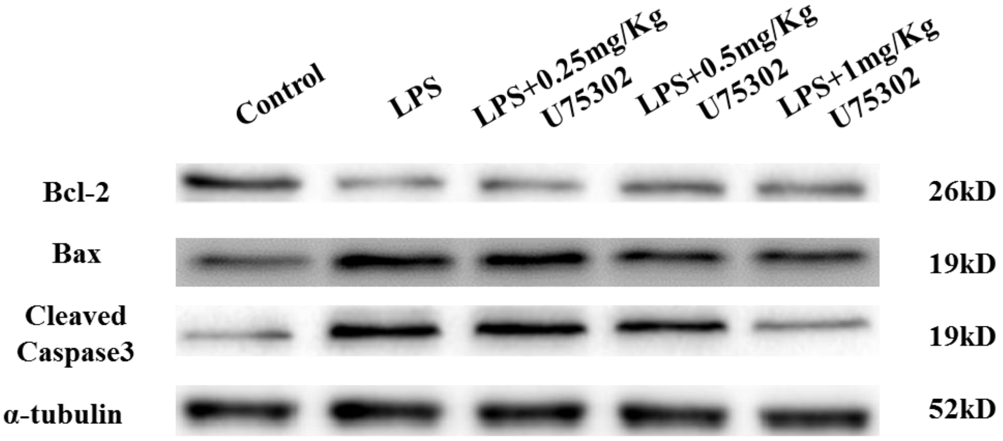

Supplementary Fig. 6 The full length blots in Fig. 5A

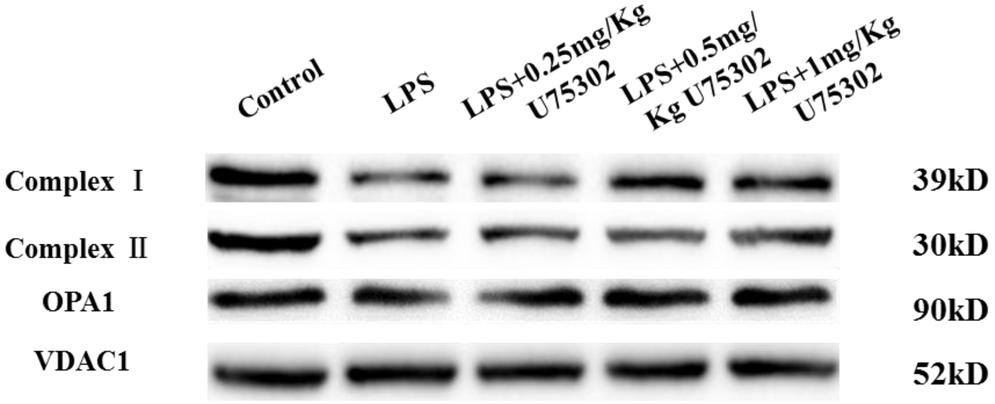

Supplementary Fig. 7 The full length blots in Fig. 8A

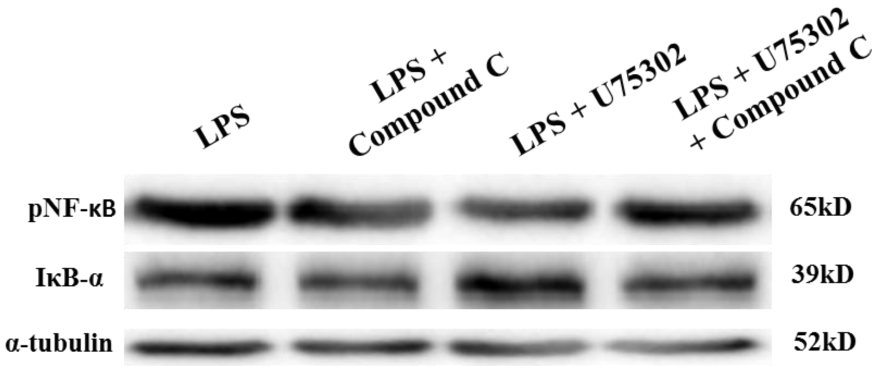

Supplementary Fig. 8 The full length blots in Fig. 9A

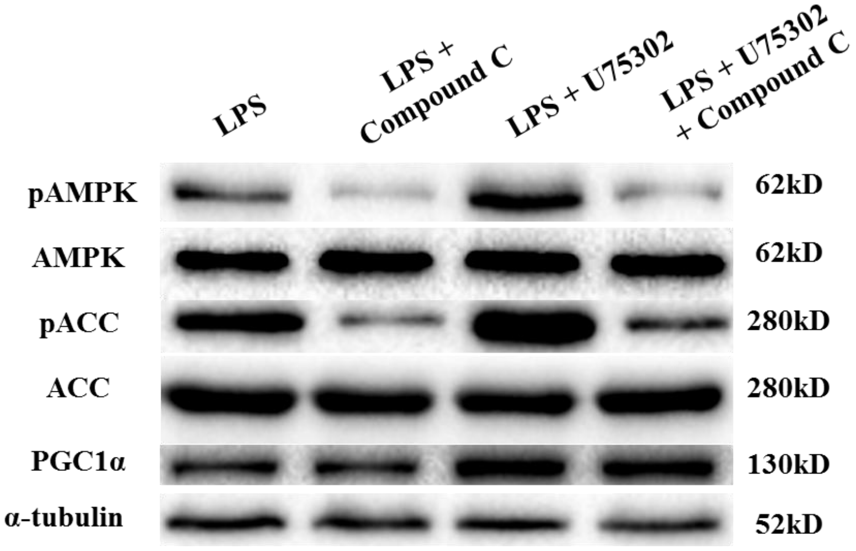

Supplementary Fig. 9 The full length blots in Fig. 10A

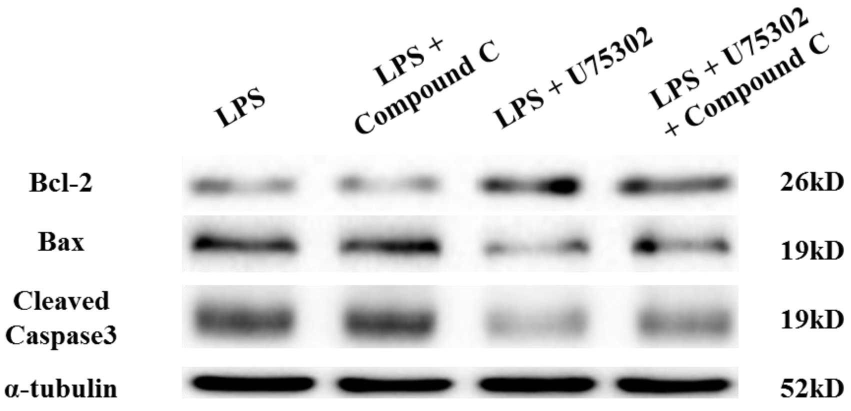

Supplementary Fig. 10 The full length blots in Fig. 11A

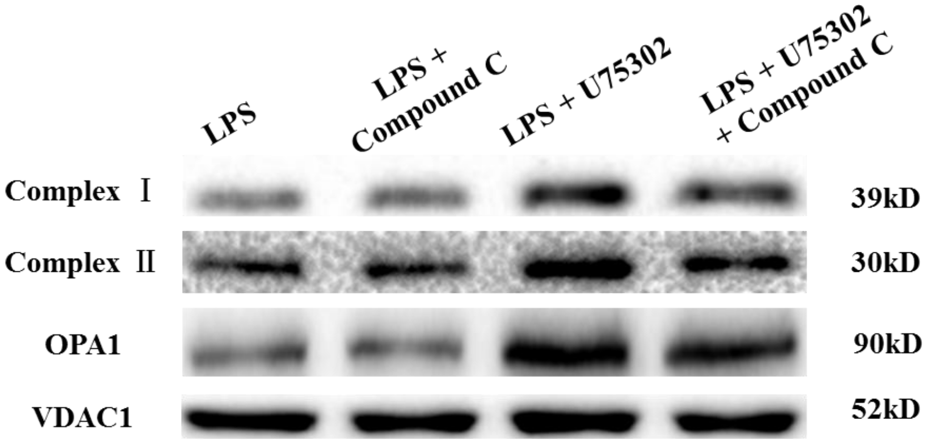

Supplementary Fig. 11 Following the editor's comments, relative experiments were conducted and representative western blot result of Bax is presented as following.

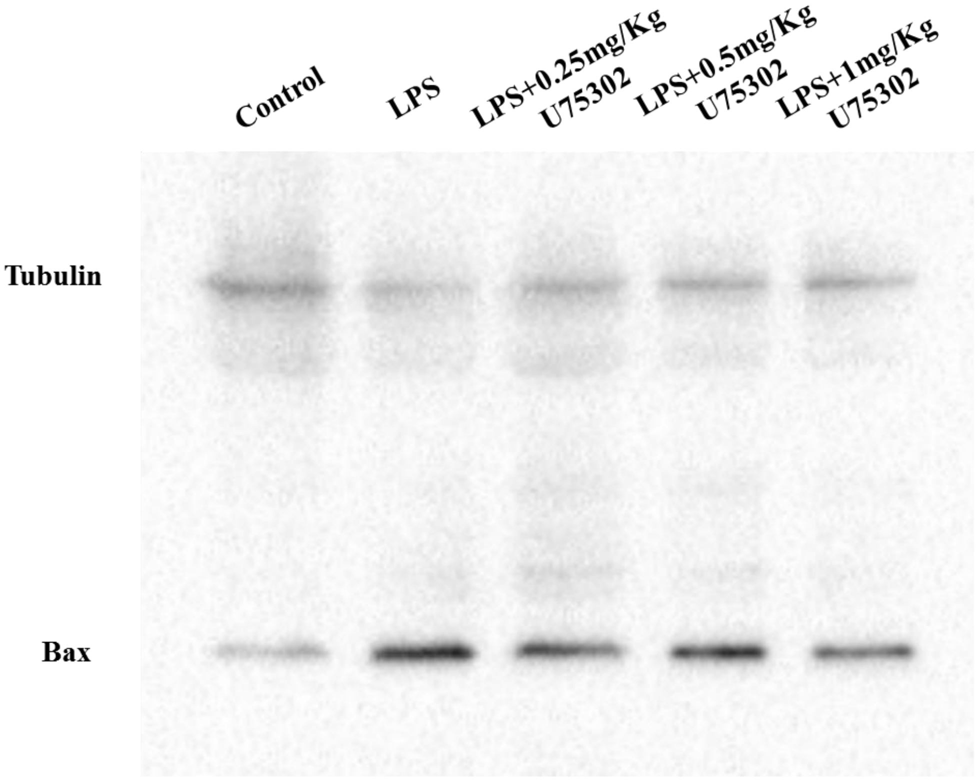

Supplement: Supplementary Information [file srep44352-s1.pdf]
